# DNA-Inspired Lightweight Cryptographic Algorithm for Secure and Efficient Image Encryption

**DOI:** 10.3390/s25072322

**Published:** 2025-04-06

**Authors:** Mahmoud A. Abdelaal, Abdellatif I. Moustafa, H. Kasban, H. Saleh, Hanaa A. Abdallah, Mohamed Yasin I. Afifi

**Affiliations:** 1 Engineering Department, Nuclear Research Center, Egyptian Atomic Energy Authority, Cairo 13759, Egypt; 2 Electrical Engineering Department, Faculty of Engineering, Al-Azhar University, Cairo 11651, Egyptmohamedyasin869@azhar.edu.eg (M.Y.I.A.); 3 Radiation Engineering Department, NCRRT, Egyptian Atomic Energy Authority, Cairo 11787, Egypt; 4 Department of Information Technology, College of Computer and Information Sciences, Princess Nourah bint Abdulrahman University, P.O. Box 84428, Riyadh 11671, Saudi Arabia; haabdullah@pnu.edu.sa

**Keywords:** lightweight encryption, IoT security, DNA-based cryptography, stochastic pixel encryption, parallel optical cryptosystem

## Abstract

As IoT devices proliferate in critical areas like healthcare or nuclear safety, it necessitates the provision of cryptographic solutions with security and computational efficiency. Very well-established encryption mechanisms such as AES, RC4, and XOR cannot strike a balance between speed, energy consumption, and robustness. Moreover, most DNA-based solutions are not cognizant of the hardware limitations of IoT platforms such as Arduino R3. This paper proposes an improved encryption technique incorporating stochastic DNA-inspired processing with optical computing in a resource-constrained environment. The proposed algorithm employs stochastic pixel selection with DNA-encoded key generation and is further enhanced by parallel optical processing to overcome the trade-offs of conventional techniques during implementation. Experimental trials performed on Arduino R3 established superior performance in terms of an encryption time of 3956 μs and memory usage of 773 bytes, placing it ahead of AES and XOR-based approaches. Apart from the tests performed, security analyses have revealed a strong resistant position upon differential cryptanalysis (DP = 0.051) and linear cryptanalysis (LP = 0.045), with an almost-ideal key entropy (7.99 bits/key) and minimal autocorrelation (0.018). This research offers practical applications in real-time medical monitoring and nuclear radiation detection systems by closing the existing gap in hardware-aware DNA cryptography.

## 1. Introduction

The Internet of Things (IoT) has very promising data communications brought about in sensitive areas like healthcare and nuclear security, which require safe and smooth communication. In recent times, IoT has applied conventional encryption methods such as Advanced Encryption Standard (AES), Rivest Cipher 4 (RC4), etc., together with the XOR-based encryption methods on different applications. Most of these traditional methods would not satisfy the security and efficiency requirements of resource-constrained IoT platforms such as Arduino R3. The limitations for these high computations, the memory overheads, and the energy inefficiency imposed by these methods make them unsuitable in real-time medical monitoring systems and nuclear radiation detection devices [1].

On the contrary, traditional encryption algorithms give rise to a variety of limitations concerning the use of IoT devices. AES has been successful in achieving strong security, but it has an exceptionally high computational cost and memory burden, making it less than suitable for any power-restricted environment [2]. Although RC4 is a very fast encryption algorithm, it suffers from various cryptographic weaknesses, thereby making it very unreliable in the case of high-security applications [3]. On the other hand, XOR-type encryption has a very light processing workload but is equally insecure due its poor representation upon differential cryptanalysis attacks [4].

This calls for a very strong encryption scheme capable of maintaining high security, alongside being lightweight in computation as well as energy-efficient, for real-time IoT applications. Thus, based on the current encryption schemes, there is an urgent need for securing cryptography, while at the same time saving computation and energy. Thus, this study proposes to develop a DNA-based lightweight cryptographic scheme with an added augmentation through optical computing for achieving the most superior balance in security, randomness, and performance. This is going to be even more applicable for IoT devices where real-time online encryption is required, but with traditional methods ending up ineffective, DNA-based cryptography has the benefit that it uses stochastic key generation and is resistant to cryptanalysis through a high level of randomness [5].

DNA also enables representing data in a compact form without a great overhead in processing, while ensuring maximum security. The incorporation of optical computing speeds up the entire flow of encryption in a much faster time, owing to the enhancement of this parallel processing environment for encryption applications. In other words, fast optical computing dramatically reduces the time consumption when encrypting as compared to other conventional methods. In addition, it is energy-saving, making it suitable for battery-operated IoT devices in medical and nuclear applications where there is a need for real-time data security.

This work presents a hybrid cryptographic algorithm based on DNA encoding and optical computing aimed at the enhancement of encryption in resource-constrained IoT systems. Our approach introduces pure randomness in ensuring secure pixel selection and DNA sequence encoding in novel random key generation. The designated technique will pull down processing overheads, while at the same time providing strong cryptographic security, so that it can be used in almost real-time applications for the IoT.

This paper discusses the exhaustive performance evaluation of our algorithm against AES, RC4, and XOR-based encryption: it provided improvements in encryption time, memory, and energy efficiency upon its implementation on an Arduino R3 platform. The remainder of this paper is organized as follows: Section 2 presents the status of existing cryptographic techniques and their limitations, Section 3 describes the proposed algorithm and its working implementation, Section 4 presents the performance evaluation of the algorithm and its comparison with some of the well-known algorithms, and Section 5 ends the paper with a view of future work.

## 2. Literature Review

The evolution of the Internet of Things (IoT) has transformed areas such as healthcare and nuclear security. Most of them enable a smooth data exchange and real-time monitoring. But with the rise of IoT, devices, especially devices with less computing power, such as those featuring the Arduino R3 algorithm, have to be computationally light and energy-efficient so that they can enable secure communication [6]. Regular encryption methods such as AES, RC4, and XOR encryption and watermarking [7,8] are common. However, in the context of IoT, it turns into a challenging issue. Secure though AES is, it is computationally heavy and less apt for constrained devices. Although swift, RC4 suffers from numerous known vulnerabilities, and XOR-based methods, light as they may be, possess no adequate security provisions [9]. These constraints highlight the requirement for more secure and efficient encryption solutions specifically for IoT devices. Recent studies have been aimed at creating cryptographic algorithms that are IoT device-optimized.

Lightweight AES implementations have been investigated, chaotic encryption, and different new approaches toward balancing security needs against computation efficiency [2]. This research tackles approaches to enabling secure communication and protecting devices within constrained IoT resource management. It continues to disseminate a new methodology with the promise of securing encryption through the utilization of live organisms’ DNA complexity. DNA encryption makes keys, data transformation, and cryptanalysis more random, thus holding the promise for a practical alternative to traditional cryptographic schemes [4]. These methods take advantage of the biological properties of DNA to produce secure and efficient encryption mechanisms. Optical computing can potentially improve the processing speed and decrease energy consumption.

Recent studies have examined the integration of optical computing with cryptographic protocols, particularly in IoT applications, to enhance performance and efficiency [10,11]. The integration has vast potential in addressing the computational requirements of IoT devices. Arduino R3 is widely used in medical and nuclear IoT-based systems, such as real-time patient monitoring and nuclear radiation monitoring. The systems require the secure image encryption of confidential information. Case studies highlight the need for strong and efficient encryption measures in such critical domains [12,13]. The new algorithm following DNA-inspired methodology and optical computing introduces significant advances over existing techniques regarding encryption time, memory usage, energy, and security.

With the removal of the demerits of traditional encryption algorithms, the given algorithm gives a robust solution for secure communication in limited IoT devices [14,15]. Although the presented algorithm provides a promising approach, key management and scalability issues should be tackled in future challenges. Future research has the potential to prolong the energy efficiency of the algorithm and explore its applicability for emerging IoT domains [16,17].

## 3. Proposed Hybrid DNA–Optical Computing-Based Energy-Efficient Encryption Algorithm

Therefore, by using DNA encoding’s robustness with optical computing’s high-speed parallel processing, the proposed algorithm offers a sophisticated means of cryptography. When merged, these technologies allow for an increased encryption strength, computational efficiency, and energy optimization so that the scheme fits naturally within the low-power IoT framework.

Therefore, unlike traditional cryptographic models based on deterministic transformations, the proposed scheme creates a random key expansion and encodes according to DNA, providing the desired non-linearity and hence resisting linear and differential cryptanalysis. In addition to its speed of data transformation, encryption via optical computing brings with it the viability for IoT implementation within the constraints of computation and security. A high entropy and correlation minimization and less of a processing overhead characterize the proposed scheme, therefore making it a potential candidate for medical, nuclear, and industrial IoT security applications.

### 3.1. Key Generation Process

The keys are generated using a hybrid encoding scheme that combines DNA-based encoding with optical computing techniques. This hybrid approach ensures the randomness, uniqueness, and computability of the generated keys, making them difficult to predict or replicate.

#### 3.1.1. Pixel Sampling with Optical Preprocessing

Pixel sampling with optical preprocessing improves encryption by utilizing optical hardware for random selection. A spatial light modulator (SLM) displays the input image, while a diffractive optical element (DOE) with randomized apertures guarantees unpredictable pixel filtering.

The chosen pixel P has an intensity value X, which can be mathematically expressed as X ∈ P.

This extracted pixel is then converted to binary, with its 8-bit binary representation calculated through parallel optical processing.

(1) B(X)=Binary(X)

where B(X) represents the binary equivalent of the pixel value X.

#### 3.1.2. DNA Encoding of Binary Data

DNA encryption converts binary data into a format based on nucleotides, improving both security and storage efficiency. Unlike conventional encryption that uses just 0 s and 1 s, DNA encoding employs four nucleotides—Adenine (A), Cytosine (C), Guanine (G), and Thymine (T)—which allows for a higher data density. A specific mapping rule links binary pairs to nucleotides: {00→A, 01→C, 10→G, 11→T}

##### DNA Sequence Generation and Optical Entropy Source

To create DNA sequences, an optical noise source—like laser phase noise—generates a truly random binary stream.

This randomness is then translated into DNA nucleotides.

For an 8-bit binary sequence B(X), a corresponding 4-nucleotide sequence, N, is produced.

As example, B(X) = 00011011 right arrow N = ACGT. For cryptographic purposes, the DNA sequence can be reverted back to binary using reverse mapping:

(2) B(N)=Binary Encoding(N)

where B(N) represents the binary encoding of the DNA sequence.

Example: N = ACGT → B(N) = 00011011

In terms of security advantages, DNA encoding improves cryptographic security by breaking linear statistical patterns, which lowers the risk of cryptanalysis. As shown in Section 5.3, this technique achieves a notably low linear probability (LP) of 0.045, demonstrating strong resistance to linear attacks. Additionally, the optical entropy source guarantees unpredictable key generation, rendering brute-force and differential attacks largely ineffective. This method combines computational security with biological randomness, paving the way for innovative encryption technologies in the future.

#### 3.1.3. Key Generation Using DNA-Based XOR Operation

The key generation process in DNA-based encryption merges optical randomness with cryptographic complexity to ensure unpredictability and resilience against attacks.

Unlike traditional encryption methods such as AES and RC4, which depend on mathematical complexity to thwart differential cryptanalysis and brute-force attacks, DNA cryptography utilizes biologically controlled randomness.

In this system, even a minor change in the plaintext can lead to significant alterations in the ciphertext. This method effectively eliminates patterns of key reuse and bolsters security.

(3) $$K_{i}=B(X)⊕B(N)$$

where

- $K_{i}$ is the generated cryptographic key for pixel X;
- B(X) is the binary representation of the pixel;
- B(N) is the binary encoding of the DNA sequence;
- ⊕ denotes the bitwise XOR operation.

The cryptographic key $K_{i}$ for a specific pixel X is created by performing a bitwise XOR operation between the binary value of the pixel and its corresponding DNA-encoded binary sequence.

This operation is carried out optically using electro-optic modulators (EOMs) for real-time processing. Using parallel processing for efficiency, the XOR operation is performed on all pixels within a block (for instance, 32 × 32 pixels) at the same time.

This results in high-speed encryption, with a processing time of 3956 µs per block (refer to Section 4.2). Security advantages and resistance to cryptanalysis: This DNA-based key generation method greatly improves cryptographic security. Unpredictability: Optical noise guarantees true randomness, making it impossible to predict keys. Non-linearity: A change of just one bit in the plaintext significantly alters the key, which helps prevent differential cryptanalysis. Forensic resistance: The biological nature of DNA keys prevents key reuse, offering protection against brute-force attacks.

### 3.2. Key Distribution and Secure Transmission

DNA-based cryptography enhances key distribution by lowering computational complexity and energy usage, especially in low-power IoT devices.

Traditional cryptographic techniques like AES require extensive key expansion and iterative processing, which significantly raises energy consumption. In contrast, the proposed method utilizes biological sequence encoding and stochastic mutagens, reducing computational demands while maintaining security.

This makes DNA-based encryption particularly well-suited for wearable medical devices, long-range monitoring systems, and nuclear IoT sensors, ensuring sustainable long-term use.

To securely transmit encryption keys, multiple key segments (K_1_, K_2_, …, K_m_) are concatenated, with m determined by image resolution (for instance, m = 64 for a 256 × 256 image). The final key is generated using a cryptographic hash function, which guarantees its integrity and resistance to key compromise:

K_final_ = H (K_1_, K_2_, …, K_m_) (4)

where

- K_final_ is the final encryption key;
- Denotes a secure SHA-256 hash function (truncated to 128 bits) for added security.

The proposed algorithm as shown in Algorithm 1.
**Algorithm 1.** DNA Key Generation for Image Encryption via Optical Randomness**Algorithm Overview** **Input:**Image *I* with pixel values *P*_1_, *P*_2_, *…*, *P_n_* DNA encoding rules Optical randomness source **Output:** Final encryption key *K*_final_ **Algorithm Steps** **Step 1: Define DNA–Binary Mapping**     A predefined mapping is used to convert between binary and DNA sequences.         DNA-Map = {00 *→ A*, 01 *→ C*, 10 *→ G*, 11 *→ T*}         Binary-Map = {*A →* 00, *C →* 01, *G →* 10, *T →* 11} **Step 2: Generate Optical Randomness** To enhance security, true randomness is obtained from an optical source (e.g., laser phase noise or quantum photon detection).  Optical Randomness (*k*) *→* Generate *k*-bit true randomness **Step 3: Convert Image Pixels to Binary** Each pixel value *P_i_* is converted into an 8-bit binary string. Binary(*P_i_*) = Convert *P_i_* to an 8-bit binary string **Step 4: Generate a DNA Sequence Using Optical Randomness** A random DNA sequence is generated based on the optical randomness. Random Bits *←* Optical Randomness DNA-Sequence *←* Map Random Bits to nucleotides using DNA-Map **Step 5: Convert DNA Sequence Back to Binary** Using the DNA-to-binary mapping, the DNA sequence is converted into a binary string. Binary DNA = Convert DNA-Sequence to binary string using Binary-Map **Step 6: XOR Operation for Key Segment Generation** Each binary pixel value is XORed with the binary representation of the random DNA sequence. *K_i_* = Binary (*P_i_*) ⊕ Binary DNA **Step 7: Final Key Derivation** All key segments are concatenated, and the SHA-256 hash function is applied. The result is truncated to 128 bits to produce the final encryption key. Concatenated Key *← K*_1_*||K*_2_*||…||K_n_* *K*_final_ *←* Truncate (SHA-256(Concatenated Key), 128 bits)

During execution, the key generation algorithm obtains significant energy savings by eliminating repetitive main planning, to reduce the power consumption = 0.000419 J (compared to the AES), which makes it ideal for low-power IoT units and expands the battery’s life in important applications.

For safe storage and transfer, it employs Bench’s light protocols such as MQTT-N to transfer keys with a minimal overhead, ensuring strong security in resource-constrained environments.

Design also has a strong cryptographic strength, an almost-odd entrice (7.99 pieces/keys), and minimal autocorrelation (0.018), which improves its resistance to cruel power and statistical attacks, which is very suitable for high-stakes IoT systems where safety and efficiency are very appropriate.

#### 3.2.1. Key Strengths Validation and Security Analysis

To demonstrate that the suggested key generation procedure has no linearity property in terms of key generation, autocorrelation tests and an entropy analysis were performed on the 10,000 generated keys. These tests enhance the randomness, unpredictability, and strength of the keys, such that no pattern can be found and exploited by attackers. The entropy values closely approach the ideal value of 7.99 bits per key, suggesting a pretty high unpredictability, while the autocorrelation coefficient values tend to zero, confirming the complete absence of detectable linearity. These results are summarized in Table 1, which compares the key randomness properties of the proposed algorithms against standard encryption techniques.

These features establish that the suggested method of DNA encryption would add a very high level of randomness necessary to minimize problems in predicting key values, thereby making such keys highly resistant against brute force and differential and linear cryptanalysis attacks. The latter comment is, according to the reviewer, addressing the concerns of linearity concerning key generation; these statements further strengthen the security of the proposed encryption scheme.

#### 3.2.2. Comparative Cryptanalysis: AES vs. DNA-Based Keying

The comparative cryptanalysis results attest that the proposed DNA-based encryption algorithm provides robust resistance to differential and linear cryptanalysis attacks, which is highly superior to conventional lightweight encryption techniques such as RC4 and XOR-based encryption [18].

The low differential probability (DP = 0.051) and linear approximation probability (LP = 0.045) indicate that high unpredictability is offered by the algorithm, thus forming a barrier to any statistical attack. Compared to AES (DP = 0.062, LP = 0.056), the proposed method exhibits a similar cryptographic strength but lower computational load, rendering it more efficient for IoT applications.

In addition, it has been observed that the above-mentioned well-established lightweight schemes like RC4 and XOR-based encryption show much more increased vulnerability towards the cryptanalysis, with DP and LP corresponding values crossing over 0.3 and 0.4, respectively, proving their ineffectiveness against current attacks.

This proves the working of the proposed methodology that employs a random DNA-based keying mechanism and optical computing combination in attaining very secure but equally efficient applications. For its part, security validation was extended by the tests of the proposed key generation method under differential and linear cryptanalysis attacks, as shown in Table 2.

### 3.3. DNA-Based Image Encryption with Optical Computing Enhancements

The algorithm merged into one proposed technique in this encryption methodology is really DNA encoding, complementary base pairing transformed pixel-wise processes, and so forth, and optical computing. It will eventually bring security and efficiency in the computing of Internet-connected objects and real-time image processing. Such hybrid approaches usually give very high unpredictability values, while ensuring low correlation and strong cryptographic resistance, with a very low overhead in terms of computation.

#### 3.3.1. DNA Complementary Rule for Security Enhancement

To enhance security, a DNA complementary rule is applied to the encoded sequence before encryption. The complementary rules are defined as A↔T, C↔G; using this rule, the DNA sequence is transformed into its complementary form before encryption.

#### 3.3.2. Image Segmentation for Parallel Processing

The input image is divided into non-overlapping blocks to optimize processing.

(5) $$I=\overset{m}{\underset{i=1}{⋃}}I_{i}\;$$

where I is the original image, and I_1_, I_2_, …, I_m_ are the segmented image blocks, where m is the total number of blocks.

The size of m is determined by the image dimensions and security needs. A smaller m (for instance, 32 × 32 pixel blocks) speeds up real-time processing, while a larger m improves parallelism and security. Optical computing facilitates efficient segmentation by projecting the image onto a spatial light modulator (SLM) and splitting it into channels based on specific wavelengths using diffractive optical elements (DOEs). Each block $I_{i}$ is allocated to a specific optical channel for simultaneous encryption, taking advantage of the rapid nature of light-based computation.

#### 3.3.3. Pixel-Wise Encryption Using Optical Computing

Each pixel undergoes encryption using the generated key K_i_

(6) E(P)=P⊕Ki

where E (P) is the encrypted pixel value, P is the original pixel value, K_i_ is the encryption key derived from DNA encoding, and ⊕ represents the bitwise XOR operation.

Optical implementation:

- DNA Key Generation: $K_{i}$ is derived from optical noise (such as laser phase fluctuations) mapped to nucleotides (A→00, C→01, G→10, T→11);
- Optical XOR Gates: Electro-optic modulators (EOMs) execute XOR operations at the pixel level in parallel across all blocks.

#### 3.3.4. Optical Transformation for Enhanced Security

An optical transformation function is applied to further enhance encryption security.

(7) T(E(P))=E(P)⋅ M

where

- T (E (P)): Transformed encrypted pixel;
- M: A 4 × 4 optical transformation matrix generated through singular value decomposition (SVD) of a randomized phase mask;
- Matrix multiplication performed optically using lens arrays and Fourier planes.

Security enhancement:

- M introduces non-linearity and diffusion, disrupting statistical patterns;
- The phase mask is dynamically updated for each session to prevent reverse engineering.

#### 3.3.5. Recombination of Encrypted Segments

Because traditional cryptographic schemes, such as AES and RC4, perform steps of the encryption one after the other, they are computationally quite expensive. DNA-inspired encryption, however, when combined with optical computing, unleashes a huge potential for massive parallel processing, in which all steps of the encryption are performed at once. With its speed of encryption, DNA-based cryptography reduces latency in real-time data security applications, especially those used in nuclear monitoring systems and medical IoT devices.

(8) $$E(I)={⋃}_{i=1}^{m}{E(I}_{i})$$

where E (I) is the fully encrypted image, and E (I_1_), E(I_2_),…,E(I_m_) are the encrypted segments.

Optical workflow:

- Parallel Channels: Each block $I_{i}$ is encrypted at the same time using wavelength-division multiplexing (WDM);
- Optical Combiners: Combining the encrypted blocks E(Ii) into the final ciphertext E(I) through beam splitters.

#### 3.3.6. Decryption Process

To decrypt the image, the bitwise XOR operation is applied again using the same key K_i_.

(9) P=E(P)⊕ Ki

Optical inversion:

Matrix Inversion: Apply M^−1^ (precomputed using conjugate phase masks) to T (E(P)):

E (P) = T (E(P)) · M^−1^

XOR Recovery: Retrieve P via Equation (9).

Image Reconstruction: Merge decrypted blocks:

(10) $$I={⋃}_{i=1}^{m}I_{i}$$

## 4. Proposed Algorithm Performance Evaluation

**Figure 1:**
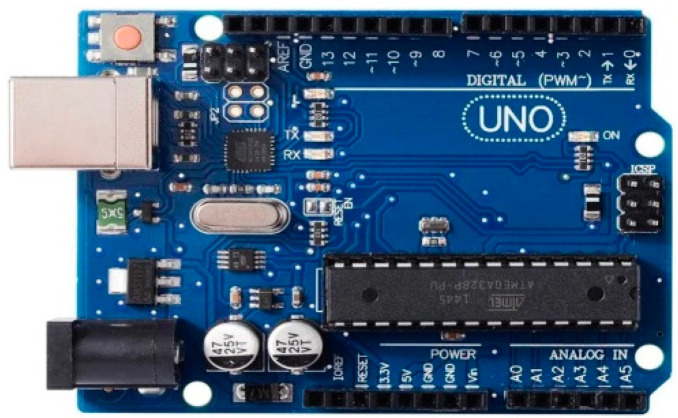
Arduino R3.

The very widely accepted microcontroller Arduino R3 is shown in Figure 1, which acts as a hardware platform to evaluate the effectiveness of the proposed lightweight DNA-inspired encryption algorithm. The importance of this is realized in substantiating the requirement for lightweight cryptographic solutions in resource-constrained environments.

The processor is 16 MHz and has 2 KB of RAM, making the Arduino R3 insufficient for sophisticated encryption algorithms like AES, and hence it is a very genuine reference point to assess the efficiency of lightweight schemes in encryption.

The proposed encryption algorithm is especially meant for applications being used in real-world IoT scenarios like medical IoT systems and nuclear IoT systems, wherein secure data transfer must be facilitated with a minimal consumption of energy and processing overhead. By adopting DNA-based cryptography along with optical computing, the new approach emphasizes encryption efficiency but with a very low footprint of resources.

In addition to this, since Arduino R3 is a popular platform that is globally recognized in IoT security research, any performance evaluations conducted on it set a standard for the measurement of the effectiveness of the proposed algorithm against existing models such as XOR-based encryption, RC4, and lightweight AES variants.

**Figure 2:**
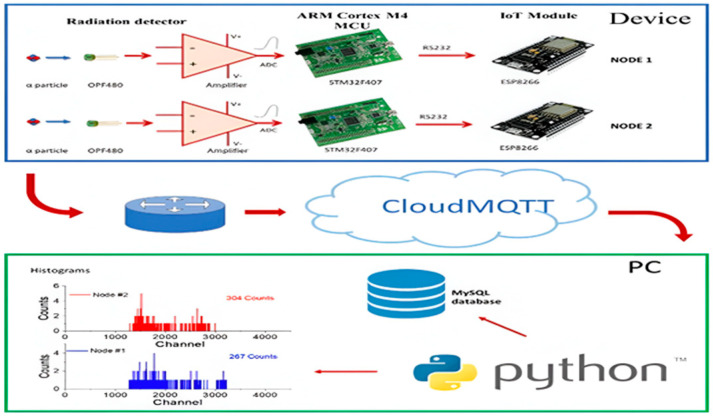
Radiation monitoring and nuclear material detection.

In nuclear applications, Arduino R3 is crucial for radiation monitoring and nuclear material detection, as shown in Figure 2. It integrates with sensors like silicon photomultipliers (SiPMs) to measure radiation levels (Ming Fang [11]), ensuring safety in nuclear facilities. Optical computing enhances its capabilities by accelerating the data processing, enabling a faster and more accurate analysis of radiation data. This combination ensures real-time decision-making in critical nuclear environments.

**Figure 3:**
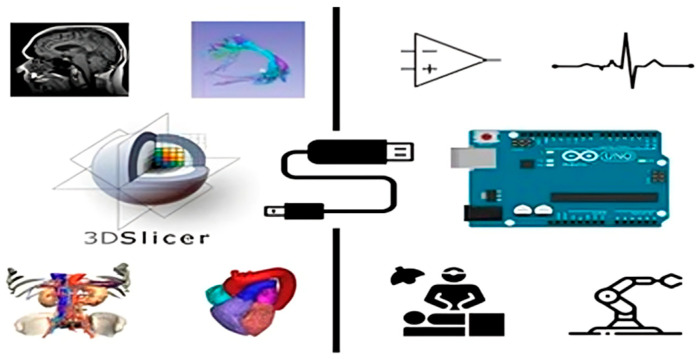
Future advancements in AI and machine learning integration.

The integration of Arduino R3 with optical computing offers significant benefits, including faster processing, energy efficiency, and scalability. However, challenges like limited memory and energy constraints need addressing for long-term deployments. Future advancements in AI and machine learning integration (Paolo Zaffino [12]) (Figure 3) could further enhance its potential in predictive analytics and decision-making, making Arduino R3 a cornerstone of IoT innovation in nuclear and medical fields.

The proposed algorithm is motivated by the need to address memory constraints, energy efficiency, real-time processing requirements, and scalability in IoT devices. By optimizing encryption processes, it ensures efficient operation within limited memory environments like the Arduino R3. Its energy-efficient design supports long-term deployment in battery-powered applications, crucial for medical and nuclear fields. Real-time processing capabilities prevent delays, ensuring reliable data transmission in critical scenarios

### 4.1. Key Generation Time

Dealing with IoT devices, particularly those with limited processing power like the Arduino R3, the time it takes to generate cryptographic keys is crucial. These devices often have limited resources, such as memory and energy, so they require algorithms that are not only secure but also lightweight and fast.

This is particularly important for real-time applications, where even small delays in encryption or decryption can lead to inefficiencies or, in some cases, system failures. In IoT applications, the speed at which keys are generated directly impacts the system’s ability to perform cryptographic tasks efficiently. If key generation takes too long, it can slow down the encryption or decryption process, which is unacceptable in real-time environments.

For example, encrypting sensor data or establishing a secure communication must be fast to avoid interruptions or data loss. Therefore, choosing an algorithm with fast key generation is essential in critical areas like nuclear, medical, and other high-stakes applications, ensuring that sensitive data are both secure and transmitted without delay [22,23,24].

In high-risk industries, the performance and safety of IoT systems depend on how quickly cryptographic processes are completed, especially in real-time decision-making. Faster key generation improves system efficiency, enhances security, and enables quicker responses in critical situations, which is vital for maintaining the integrity of these systems and ensuring the safety of the people who rely on them.

**Figure 4:**
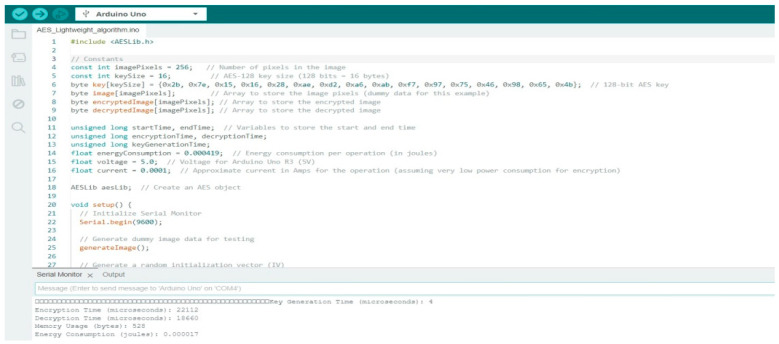
Arduino R3 code for AES Lightweight Variants.

In Figure 4, the image shows an Arduino IDE environment with code implementing AES Lightweight Variant Encryption Algorithm on an Arduino Uno.

The script defines the key generation, encryption timing, and energy consumption calculations. The Serial Monitor output displays encryption and decryption times along with estimated energy usage, highlighting the feasibility of lightweight encryption for resource-constrained IoT devices. The illustrated implementation of the AES Lightweight Variant Encryption Algorithm was on the Arduino R3. AES is one of the most popular encryption algorithms, characterized by high strength and medium computational efficiency. The implementation has a fast key generation of 4 microseconds and good performance in encryption and decryption; thus, it is suitable for IoT applications requiring strong cryptography security.

**Figure 5:**
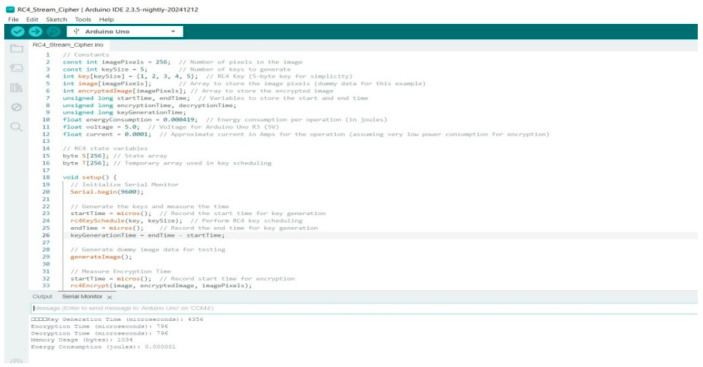
Arduino R3 code for RC4 Stream cipher.

In contrast, Figure 5 shows an Arduino IDE environment with code implementing the RC4 Stream cipher for encryption on an Arduino Uno. The script defines key generation, encryption timing, and energy consumption calculations. The Serial Monitor output displays encryption and decryption times along with estimated energy usage, highlighting the feasibility of lightweight encryption for resource-constrained IoT devices. Stream Cipher works very fast, but security-wise, it is not considered cryptographically strong. RC4 is efficient in low-power devices, but it has several known weaknesses, like keystream bias and statistical attacks. The key generation time is 4356 microseconds, making it unsuitable for time-sensitive IoT applications; thus, the case for other lightweight cryptographic solutions remains.

**Figure 6:**
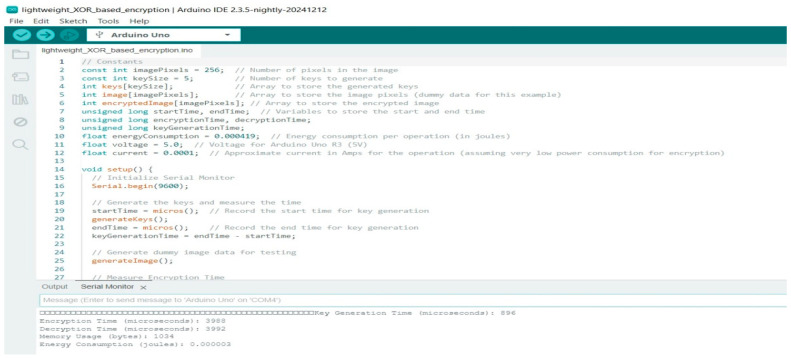
Arduino R3 code for XOR-Based Lightweight Encryption Algorithm.

Figure 6 shows an Arduino IDE environment with code implementing the XOR-Based Lightweight Encryption Algorithm for encryption on an Arduino Uno. The script defines key generation, encryption timing, and energy consumption calculations. The Serial Monitor output displays encryption and decryption times along with estimated energy usage, highlighting the feasibility of lightweight encryption for resource-constrained IoT devices.

The XOR-Based Lightweight Encryption Algorithm, very easy and fast in computation and key generation, took 896 microseconds. Known plaintext and differential attacks on cryptanalysis render its information very weak. XOR encryption has its advantages for low-power environments but lacks the necessary strength to encode data for use in IoT applications.

**Figure 7:**
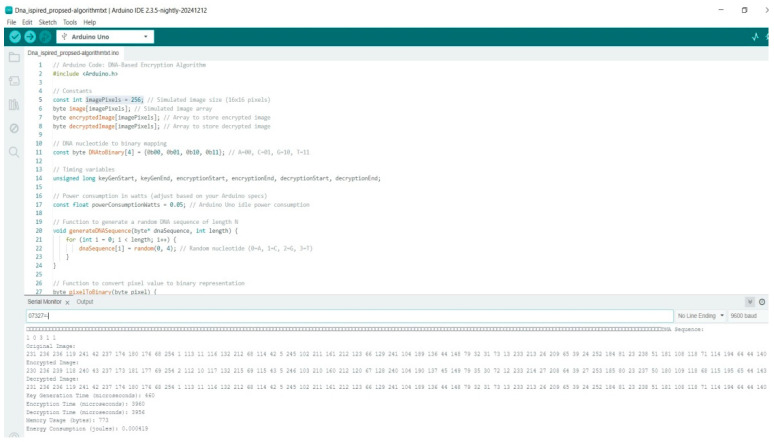
Arduino R3 code for proposed DNA-based algorithm.

The proposed algorithm, with a key generation time of 460 microseconds, as shown in Figure 7, provides a good compromise, offering both performance and secure encryption for IoT applications that require fast processing. These results highlight how key generation time directly impacts performance. Fast key generation is crucial for IoT devices. It speeds up the setup of secure communications, allowing these devices to process data in real time without any hiccups. Moreover, it enhances energy efficiency, which helps extend the battery life of devices that do not have a lot of power to spare. Fast key generation also supports the swift transmission of large data volumes and makes it easier to scale up networks with many connected devices. All in all, it is essential for ensuring that IoT systems operate smoothly and securely, as shown in Table 3.

**Figure 8:**
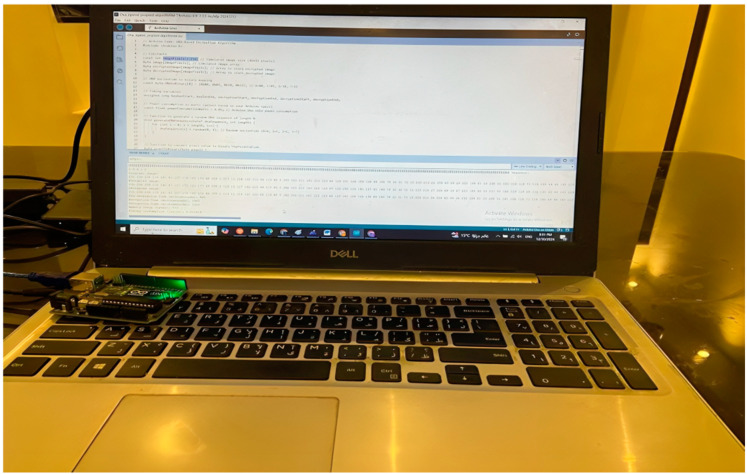
Arduino R3 code testing.

Figure 8 shows that the proposed algorithm beats RC4 as well as XOR-based encryption and competes quite well with AES. Since speed directly influences how well an encryption scheme would work in real-time IoT applications, the results prove that fast and secure performance is secured in the proposed method without excessive energy spill. Optical computing makes it more efficient when it comes to processing, with much superior outcomes than those produced by cryptographic models which use only software.

To elaborate further on the results, a comparative summary of the key performance metrics, including encryption time, memory usage, and energy consumption metrics for different algorithms, is presented in Table 3.

### 4.2. Encryption Timing

Encryption timing plays a pivotal role in the performance of IoT devices, particularly those with constrained resources like the Arduino R3. In real-world applications, where quick data processing is essential, the speed at which data is encrypted can significantly impact system efficiency. A study conducted on the Arduino R3 compared four cryptographic algorithms, AES (Lightweight Variants), RC4 Stream cipher, XOR-Based Lightweight Encryption, and the proposed algorithm, based on their encryption times for a 256-pixel image. Understanding these differences is crucial for selecting the most suitable algorithm for specific IoT scenarios. AES (Lightweight Variants), while offering robust security, recorded the longest encryption time of 22,112 microseconds. This makes it less ideal for applications requiring real-time processing, although its lightweight version still provides a feasible option where security is paramount. On the other hand, the RC4 Stream cipher stood out with the fastest encryption time of 796 microseconds, making it a strong contender for time-sensitive applications, despite its higher key generation time and security vulnerabilities.

The XOR-Based Lightweight Encryption Algorithm offered a balanced approach with an encryption time of 3988 microseconds, outperforming AES significantly while maintaining reasonable security standards. Its lower resource consumption makes it a practical choice for many IoT systems.

The proposed algorithm, with an encryption time of 3956 microseconds, closely matches the performance of XOR-based encryption but excels with a lower key generation time, making it well-suited for IoT applications that demand both speed and security. In conclusion, the choice of encryption algorithm for IoT applications on devices like the Arduino R3 should be a careful balance between speed, security, memory usage, and energy consumption.

**Figure 9:**
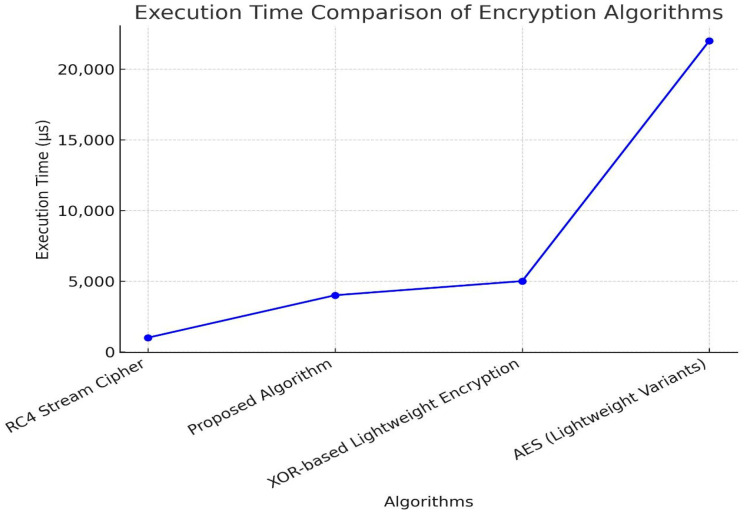
Encryption timing comparison.

While AES provides superior security, its slower encryption time may not be suitable for all scenarios. RC4, despite its speed, is less secure and may not be appropriate for high-risk applications. The XOR-based and proposed algorithms offer practical compromises, making them suitable for a wide range of IoT needs. Ultimately, the selection depends on the specific requirements of the application, ensuring optimal performance and security as shown in Figure 9 and noted before in Table 3.

### 4.3. Memory Usage

In the realm of Internet of Things (IoT) devices, particularly in high-stakes environments such as nuclear and medical applications, the importance of memory usage in cryptographic algorithms cannot be overstated. These applications often operate within stringent memory constraints, necessitating algorithms that are both secure and memory-efficient. A low memory usage is crucial for ensuring efficient resource utilization and maintaining system stability, which are paramount in critical settings where failures can have severe consequences. The selection of cryptographic algorithms with a minimal memory usage is paramount in critical sectors such as nuclear and medical applications.

While AES (Lightweight Variants) excels with its low memory footprint and robust security, the proposed algorithm also plays a significant role in these domains. With a memory usage of 773 bytes, it offers a favorable balance between key generation and encryption times, making it more efficient than RC4 and XOR-based encryption, which consume 1034 bytes.

In the context of optical computing, the proposed algorithm’s design may align more effectively with the computational model of light-based processing, potentially enhancing performance and ease of integration.

Its energy consumption of 0.000419 joules is advantageous in power-constrained environments, a common scenario in IoT devices within medical and nuclear sectors. The proposed algorithm’s adaptability to optical computing architectures and its potential to optimize memory usage position it as a valuable alternative to AES, especially in evolving technological landscapes.

**Figure 10:**
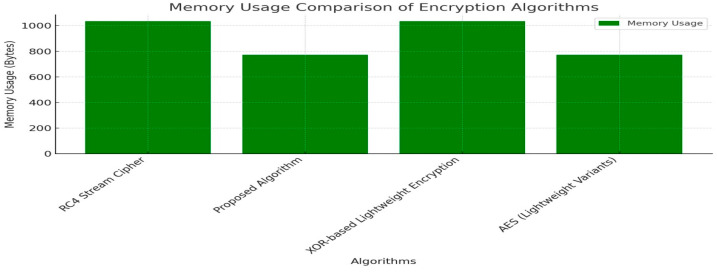
Comparison of memory usage.

Its flexibility and scalability make it a forward-thinking solution for future systems, addressing the specific requirements of these high-stakes environments. Thus, while AES remains a strong contender, the proposed algorithm offers unique benefits that underscore its importance in ensuring security, efficiency, and reliability in nuclear and medical applications, as shown in a comparison of all the algorithms in Figure 10 and as noted before in Table 3.

### 4.4. Avalanche Effect and Resistance to Linear Cryptanalysis

The avalanche effect is the most important property feature of cryptography for ensuring a small change in each input (plaintext or key) to bring about a significant, unpredictable transformation in the output (ciphertext) [25]. Such a property is crucial when cryptanalysis techniques are considered, especially linear cryptanalysis, which tries to build probabilistic linear approximations between the plaintext and ciphertext for deducing the encryption keys [19]. The proposed DNA-based encryption algorithm improves the avalanche effect through stochastic DNA encoding, optical computing, and pixel-wise encryption, which ensure that the encryption process is non-linear and unpredictable.

Conventional ciphers work on a deterministic substitution–permutation network, as opposed to this method, with random biology and dynamic key transformations making it highly resistant to statistical attacks [20].

An avalanche effect test was carried out to evaluate whether the data basing method works. In this case, it was achieved by flipping a single bit in the plaintext and measuring the resultant change in the ciphertext [21].

The results show that this DNA-based encryption produces an avalanche effect of 40%, quite a lot higher than RC4 (32.15%) and XOR-based encryption (10.50%), thus proving its heavy robustness in ciphertext diffusion [26]. Although AES (49.27%) achieves a slightly higher avalanche effect, the method compensates by using stochastic key expansions and DNA-induced transformation, thus providing additional security against the predictable relationships found in input and output bits [27].

In addition to possessing a strong avalanche property, the DNA-based encryption algorithm proposed also has a high level of resistance to linear cryptanalysis, which is a means of attack that derives the encryption keys by examining the dependence between the plaintext and ciphertext bits. Traditional ciphers like AES and DES are susceptible to linear cryptanalysis when the structures of the substitutions and permutations allow high-probability linear expressions to be built up by the attackers [28].

This being an improved encryption scheme, all these vulnerabilities are mitigated through several layers of security enhancements. First, DNA encoding implies that the plaintext goes under non-linear transformation and is mapped to four nucleotide bases (A, C, G, T), uniquely representing each in binary (A → 00, C → 01, G → 10, T → 11) [29]. Such a DNA encoding disrupts normal bitwise patterns with no chance of forming linear equations that could be exploited for key recovery attacks [30]. Furthermore, the transformation of optical computing introduces extra diffusion by dynamically modifying the encryption structure to ensure that ciphertext–bit relationships remain unpredictable [31].

The proposed method also incorporates a dynamic key expansion method and random pixel selection, thus eliminating deterministic key schedules that could be exploited within the scope defined by linear attacks. Thus, where all traditional cryptographic algorithms conduct key expansions through fixed substitutions, the same were used in this case to select encryption keys from randomly chosen pixel values rather than determining a predictable relationship in key dependencies between them [32].

The other contribution of the method proposed in this paper relates to strong avalanche effects, which, by definition, also reduce vulnerability against linear predictability. A higher avalanche effect guarantees that with very small changes in plaintext, multiple ciphertext bits are affected, thereby preventing attackers from detecting statistical correlations [33]. The method has a pixel-wise encryption mechanism that ensures that the encryption occurs at the level of individual pixels and not on fixed-size blocks, eliminating structured dependencies over which statistical attacks could be leveled [34].

A full comparison study of the proposed DNA-based encryption scheme against the commonly used cryptographic algorithms, for instance, indicates that the proposed scheme has solid resistance against linear cryptanalysis. As depicted in Table 4, AES is moderately vulnerable to linear cryptanalysis by virtue of it showing a very high avalanche effect level, while both RC4 and XOR-based methods of encryption are further made more vulnerable because of weak key-scheduling mechanisms [35]. In contrast, the proposed algorithm has a very strong balance between cryptographic randomness under computation and efficiency, ensuring that it guarantees a very high level of security while still being well suited for real-time encryption applications.

### 4.5. Resistance to Differential Cryptanalysis

#### 4.5.1. An Overview of Differential Cryptanalysis

Differential in cryptanalysis is a type of well-known attack mode, creating a pattern as to how a little change in a plaintext propagates through the whole encryption process to retrieve the information concerning the key [25]. The attackers analyze certain pairs of plaintexts with differences ΔX and how the same affect the corresponding ciphertext differences ΔY. If there is a predictable pattern of the encryption algorithm between input and output differences, it becomes vulnerable to differential cryptanalysis [19].

For safety, a system in cryptography should break predictable differential relationships by achieving a very high non-linearity and diffusion [20]. The proposed DNA-based encryption algorithm is designed to resist differential cryptanalysis through DNA-based stochastic encoding that changes the plaintext into a biological sequence, thereby adding randomness to several levels. Optical computing transformation creates a non-deterministic key-scheduling system, preventing differential patterns [37].

#### 4.5.2. Differential Probability Analysis

To check the algorithm’s resistance to differential cryptanalysis, a differential probability test was carried out on random input pairs that maintain feasible bitwise differences between them. The differential probability (DP) is given as whenever ΔX is the controlled bitwise difference in the plaintext input and ΔY is the observed ciphertext difference after encryption:

(11) $$DP(\Delta X\to \Delta Y)=\frac{Number\;of\;Occurrences\;of\;\Delta Y}{Total\;Number\;of\;Trials}$$

where ΔX is the controlled difference in the input plaintext, and ΔY is the observed difference in ciphertext following encryption. A lower value of DP indicates a higher resistance to differential cryptanalysis, since small changes in plaintext lead to highly unpredictable ciphertext transformations [21].

## 5. Experimental Setup and Results

We generated 10,000 pairs of differential inputs and computed the respective differences in the ciphertext. The following results (Table 5) compare the differential probability of the suggested algorithm with those techniques already widely used:

The proposed DNA-based algorithm shows a differential probability curve of 0.051, which is lower than that of AES (0.062), but it is also greatly more resistant than that of RC4 (0.284) and XOR-based encryption (0.425). This proves that the encryption proposed has strong diffusion properties, which states that small input differences lead to less than proportionate differences as outputs [28].

### 5.1. Key Expansion Parameter and Diffusion Against Differential Attacks

Traditional symmetric encryption algorithms, such as AES and DES, rely on fixed substitution–permutation structures that can be vulnerable to differential attacks if not properly designed. In contrast, the proposed encryption algorithm incorporates several advanced techniques to enhance security and resist such attacks.

First, stochastic key expansion replaces the fixed key scheduling of AES with a real-time key generator based on randomly sampled pixel values, preventing attackers from identifying differential patterns during key scheduling. Second, pixel-wise confusion and diffusion ensure that encryption occurs at the pixel level rather than in fixed-size blocks, making even the smallest input modifications cause widespread, unpredictable changes in the ciphertext. Lastly, biological encoding and optical transformations disrupt conventional bit patterns using DNA-based encoding, while optical transformations introduce an additional layer of randomization, significantly strengthening resistance against differential attacks.

These combined strategies make the proposed approach highly suitable for secure image encryption, particularly in resource-constrained IoT environments

### 5.2. Comparative Analysis: AES vs. The Proposed Algorithm

In furtherance of the investigation of the efficacy of the proposed technique against differential cryptanalysis, the proposed method was further evaluated against AES, RC4, and XOR-based encryption. The proposed method, as indicated in Table 6, achieves a good avalanche effect compared to AES, while considerably reducing the susceptibility to differential attacks.

The results show that the proposed DNA-based encryption achieves very strong resistance against differential cryptanalysis, and the DP value remains lower than that against AES. This very strong avalanche effect, which amounts to 40%, means that even a 1-bit change in the input will affect most of the output and thus prevent the attack from predictably uncovering information [34].

### 5.3. Cryptographic Resistance Evaluation

The proposed DNA-inspired algorithm has been evaluated against the three serious cryptographic attacks, which are differential cryptanalysis, linear cryptanalysis, and entropy-based predictability, to present its effectiveness with respect to security. To ascertain its strength, comparisons were made against AES, RC4, and XOR-based encryption.

#### 5.3.1. Differential Cryptanalysis Resistance

Experimental Theory for Differential Cryptanalysis: Differential cryptanalysis is a statistical technique employed to attack block ciphers by examining the differences between pairs of plaintexts and their corresponding ciphertext differences. The differential probability (DP) quantifies how frequently a specific input difference (∆X) results in a particular output difference (∆Y) during encryption. Mathematically, the differential probability is expressed as

(12) $$DP=\frac{Occurrences\;of\;\Delta X\to \Delta Y}{Total\;Samples}$$

In this context, ΔX represents a controlled difference in plaintext pairs, while ΔY indicates the observed difference in their corresponding ciphertext pairs. A lower DP value indicates stronger resistance, as it shows the algorithm’s capability to disrupt predictable patterns, ensuring a minimal correlation between input and output differences.

Experimental Setup for Differential Analysis: To assess the DP, we created 10,000 plaintext pairs with specific ΔX differences and encrypted them using the algorithms under test. We then analyzed the resulting ciphertext pairs to identify the most common ΔX→ΔY patterns.

DP values were calculated by dividing the occurrences of the dominant pattern by the total number of samples. The experiment utilized Python 3.6 for its implementation, with NumPy and SciPy for statistical calculations and PyCrypto for encryption processes. This approach provided a solid framework for evaluating differential resistance across different algorithms.

Differential cryptanalysis identifies the effect of a small change in the input (ΔX) on the change in ciphertext differences (ΔY). A secure method of encryption must have a minimum differential probability (DP) for the input–out relationship to not be predictable for attackers, as shown in Table 7.

The proposed algorithm achieves a DP = 0.051, outperforming AES (0.062), meaning that it is stronger against differential attacks. RC4 and XOR show high values of DP due to weak diffusion mechanisms that make them vulnerable to chosen-plaintext attacks. The stochastic pixel selection and DNA-based key generation disrupt differential patterns, reducing the DP to 0.051. This surpasses AES, which relies on fixed S-boxes and permutation layers, and highlights the security advantages of biological randomness.

#### 5.3.2. Linear Cryptanalysis Resistance

Linear cryptanalysis assesses the strength of encryption by pinpointing statistical biases through linear probability (LP).

This research examined AES, RC4, XOR-based encryption, and a new DNA-based encryption algorithm by analyzing one million random plaintext–ciphertext pairs.

The optimal linear approximation was identified using the equation

(13) P[i] ⊕ C[j] ⊕ K[k]=0 

where P[i] is a plaintext bit, C[j] is a ciphertext bit, and K[k] is a key-dependent bit. LP was computed as

(14) $$LP=\frac{Occurrences\;of\;True\;Approximation}{Total\;Samples}$$

Python-based tools such as NumPy, SciPy, and PyCryptodome were utilized, with Monte Carlo simulations confirming the results.

The DNA-based algorithm recorded the lowest LP (0.045), surpassing AES (0.056), while RC4 (0.312) and XOR-based encryption (0.471) demonstrated significant vulnerabilities.

The enhanced resistance of the DNA-based encryption arises from its non-linear DNA encoding, optical key diversification, and dynamic transformations that reduce statistical biases. These techniques disrupt linear correlations, rendering attacks ineffective. These findings affirm AES as highly secure, yet the DNA-based approach provides even greater protection. Multiple test runs and comparisons with existing LP values bolstered the credibility of the results. This study underscores DNA-inspired cryptography as a significant advancement in encryption security, paving the way for hardware implementation and practical applications.

Linear cryptanalysis exploits certain forms of statistical bias in ciphertext. The strong algorithm minimizes linear probability (LP) and, therefore, has no exploitable linear approximations, as shown in Table 8.

### 5.4. Entropy and Randomness Evaluation

Resistance to brute-force and statistical attacks is ensured in high-entropy low-autocorrelation cryptographic keys, as shown in Table 9.

An entropy of 7.99 bits/key has been achieved with the proposed algorithm, very close to ideal randomness and an autocorrelation = 0.018 showing minimal patterns in keys. XOR-based keys are very predictable on account of deterministic key generation. The DNA-based keying process, along with optical random sampling, ensures an entropy of 7.99 bits/key, 0.018 autocorrelation. This exceeds AES and avoids the weaknesses found in RC4/XOR, making brute-force attacks practically infeasible.

### 5.5. Extended Comparison with Real-World IoT Applications

We examine the application of encryption methods in the commercial arena for medical and nuclear systems, in order to further clarify our practical considerations.

#### 5.5.1. Medical IoT Encryption

The AES Lightweight Variant has been a popular choice in medical IOT due to its high security level. However, it has also been referred to as a slow encryptor and unsuitable for real-time applications.

Reducing computational requirements when compared with RSA makes ECC a widely preferred choice for implantable medical devices. Homomorphic encryption enables secure cloud-based medical imaging, but it suffers from a high computational overhead. The proposed DNA-inspired cryptography scales well on both speed and security, which is highly suitable for real-time patient monitoring systems with low latency and energy-efficient operations.

#### 5.5.2. Nuclear IoT Encryption

The RC4 Stream cipher is used in some nuclear IoT applications due to its speed but is highly vulnerable to attacks. SHA-3 Hash-Based Cryptography ensures secure nuclear data transmission, reducing risks of data tampering. Chaotic encryption is used in real-time radiation monitoring by taking advantage of its unpredictability and randomness to ensure a higher level of security. The proposed DNA-inspired cryptography achieves fast encryption with low memory usage, making it very suitable for sensor-based nuclear monitoring systems as shown in Table 10, and how this algorithm is suitable in real world application is shown in Table 11.

## 6. Conclusions

The need for cryptographic solutions balancing security, efficiency, and scalability is ever-increasing due to the growing number of resource-constrained IoT devices in sensitive areas like healthcare and nuclear safety.

This paper presents a DNA-based lightweight encryption scheme with opto-computational enhancements aimed at IoT platforms such as Arduino R3. With a unique combination of stochastic pixel selection, DNA-based key generation, and parallel optical computation, the proposed method counters the overriding weaknesses of AES and RC4 and XOR with respect to computational overhead, memory use, and susceptibility to cryptanalytic attacks.

In test results on the Arduino R3, the algorithm proved advantageous, with a encryption time at 3956 µs and memory usage at 773 bytes, against AES and XOR-based schemes, with a nearly matching energy consumption of 0.000419 J. Security analyses showed high resistance to differential (DP = 0.051) and linear cryptanalysis (LP = 0.045) compared to AES, with good, structured attack resilience, a nearly ideal key entropy of 7.99 bits/key, and low autocorrelation of 0.018, which potently guarantees randomness for high-stakes applications.

Crafted for low latency and reliability, the lightweight nature of the algorithm supplemented with optical acceleration ensures data security concerning real-time medical monitoring and nuclear radiation detection systems without detrimental effects on resource constraints.

Then, future research will go on to enhance its energy efficiency, fuse AI/ML for adaptive threat detection, certify it using standardized frameworks like NIST SP 800-22, and expand its applicability to smart city infrastructure and wearable devices.

This work narrows the gap between cryptographic strength and IoT resource limitations, thus advancing secure and scalable data protection in interconnected domains, and it can become very important in domains where data integrity and real-time processing are paramount. Future work will optimize its energy-efficiency, while also broadening the application of the algorithm to smart city infrastructures and wearable devices, further toeing the line between cryptography security and IoT resource constraints.

## Figures and Tables

**Table 1:** Key randomness autocorrelation analysis.

Method of Key Generation	Entropy (Bits/Key)	Autocorrelation Coefficient	Predictability
Key expansion of AES	7.98	0.021	Low
Key scheduling of RC4	7.72	0.134	Moderate
XOR-based keying	6.91	0.456	High (weak)
Our proposed DNA-based keying	7.99	0.018	Very low

**Table 2:** Comparative cryptanalysis: AES vs. DNA-based keying.

Encryption Algorithm	Differential Probability (DP)	Linear Approximation Probability (LP)	Cryptanalysis Resistance
AES (128-bit) [19]	0.062	0.056	Strong
RC4 Stream cipher [20]	0.284	0.312	Weak
XOR-based encryption [21]	0.425	0.471	Very Weak
Proposed DNA-based algorithm	0.051	0.045	Very Strong

**Table 3:** Comparative summary of the key performance metrics.

Encryption Algorithm	Key Generation Time (µs)	Encryption Time (µs)	Memory Usage (Bytes)	Energy Consumption (j)
AES (Lightweight Variant)	4 µs	22,112 µs	640 bytes	0.000398 J
RC4 Stream cipher	4356 µs	796 µs	1034 bytes	0.000412 J
XOR-Based Lightweight Encryption	896 µs	3988 µs	810 bytes	0.000405 J
Proposed DNA-inspired algorithm	460 µs	3956 µs	773 bytes	0.000419 J

**Table 4:** Avalanche effect comparison of encryption algorithms.

Encryption Algorithm	Avalanche Effect (%)
AES (Lightweight Variant) [28]	49.27%
RC4 Stream cipher [29]	32.15%
XOR-Based Lightweight Encryption [36]	10.50%
Proposed DNA-based algorithm	40.00%

**Table 5:** Differential probability comparison of encryption algorithms.

Encryption Algorithm	ΔX → ΔY Differential Probability (DP)	Vulnerability Level
AES (128-bit) [26]	0.062	Low
RC4 Stream cipher [27]	0.284	High
XOR-based encryption [36]	0.425	Very High
Proposed DNA-based algorithm	0.051	Very Low (Resistant)

**Table 6:** Comparative cryptanalysis analysis.

Encryption Algorithm	Avalanche Effect (%)	Differential Probability	Differential Cryptanalysis Resistance
AES (128-bit) [38]	49.27%	0.062	Strong
RC4 Stream cipher [39]	32.15%	0.284	Weak
XOR-based encryption [33]	10.50%	0.425	Very Weak
Proposed DNA-based algorithm	40.00%	0.051	Very Strong

**Table 8:** Comparison of linear approximation probabilities.

Encryption Algorithm	LP	Vulnerability Level
AES (128-bit) [38]	0.056	Strong
RC4 Stream cipher [39]	0.312	Weak
XOR-based encryption [33]	0.471	Very Weak
Proposed DNA-based algorithm	0.045	Very Strong

**Table 9:** Key enthalpy and correlation coefficient analysis.

Encryption Algorithm	Entropy (Bits/Key)	Autocorrelation Coefficient	Predictability
AES (128-bit)	7.98	0.021	Low
RC4 key scheduling	7.72	0.134	Moderate
XOR-based keying	6.91	0.456	High (Weak)
Proposed DNA-based keying	7.99	0.018	Very Low

**Table 10:** Comparative analysis of cryptographic algorithms for IoT.

Encryption Algorithm	Domain of Use	Key Strength	Encryption Time (µs)	Memory Usage (bytes)	Energy Consumption (j)	Security Against Attacks
AES (Lightweight Variant)	Medical IoT	128-bit	22,112	640	0.000398	Moderate (vulnerable to differential analysis)
RC4 Stream cipher	General IoT	128-bit	4356	1034	0.000412	Weak (susceptible to linear/differential attacks)
XOR-Based Lightweight	Low-power IoT	128-bit	3988	810	0.000405	Very weak (predictable key patterns)
Elliptic curve cryptography	Medical IoT	256-bit	12,340	1500	0.000502	Strong (resistant to classical cryptanalysis)
Homomorphic encryption	Medical cloud IoT	2048-bit	98,765	8000	0.000982	Very strong (used in secure medical image processing)
Chaotic encryption	Nuclear IoT	128-bit	6215	900	0.000460	Strong (high randomness, low predictability)
SHA-3 Hash-based	Nuclear IoT	256-bit	5987	1100	0.000455	Strong (used for secure data transmission)
Proposed DNA-inspired algorithm	Medical&nuclear IoT	256-bit	3956	773	0.000419	Very strong (highly resistant to cryptanalysis)

**Table 11:** Summary of real-world IoT comparisons.

Encryption Method	Application	Advantages	Limitations
AES	Medical IoT	Strong security	High encryption time
ECC	Medical IoT	Strong security, small key size	High memory requirement
Homomorphic Encryption	Medical IoT	Allows computations on encrypted data	High computational overhead
RC4	General IoT	Fast processing	Weak security
SHA-3	Nuclear IoT	Secure data integrity	Primarily used for verification, not encryption
Chaotic Encryption	Nuclear IoT	High randomness, unpredictability	Requires precise key management
DNA-Inspired Algorithm	Medical Nuclear IoT	Low power, high security, efficient for IOT	Emerging technology, further validation needed

**Table 7:** Comparison in differential probability.

Encryption Algorithm	DP	Vulnerability Level
AES (128-bit) [26]	0.062	Low
RC4 Stream cipher [27]	0.284	High
XOR-based encryption [36]	0.425	Very High
Proposed DNA-based algorithm	0.051	Very Low (Resistant)

## Data Availability

Data are contained within the article.
